# Gram-Negative Bacilli Blood Stream Infection in Patients with Severe Burns: Microbiological and Clinical Evidence from a 9-Year Cohort

**DOI:** 10.3390/ijms251910458

**Published:** 2024-09-28

**Authors:** María Fernanda Fuentes-González, Diana Fernández-Rodríguez, Claudia A. Colín-Castro, Melissa Hernández-Durán, Luis Esaú López-Jácome, Rafael Franco-Cendejas

**Affiliations:** 1 Infectious Diseases and Epidemiology Department, National Institute of Respiratory Diseases, Mexico City 14080, Mexico; dra.fugomaf@gmail.com; 2 Clinical Microbiology Laboratory, Infectious Diseases Division, National Institute of Rehabilitation “Luis Guillermo Ibarra Ibarra”, Mexico City 14389, Mexico; dianafernandezn@hotmail.com (D.F.-R.); usedat@gmail.com (C.A.C.-C.); melypsp@yahoo.com.mx (M.H.-D.); 3 Plan de Estudios Combinados en Medicina (PECEM) Facultad de Medicina, Universidad Nacional Autónoma de México, Mexico City 04510, Mexico; 4 Biology Department, Chemistry Faculty, Universidad Nacional Autónoma de México, Mexico City 04510, Mexico; 5 Biomedical Research Subdirection, National Institute of Rehabilitation “Luis Guillermo Ibarra Ibarra”, Mexico City 14389, Mexico

**Keywords:** burn, Gram-negative rods, resistance, blood cultures, antibiotics

## Abstract

Bloodstream infection is one of the most important and increasing complications in patients with severe burns. Most of the species affecting this population are Gram-negative bacilli that exhibit antimicrobial resistance. We conducted this study to determine the antimicrobial susceptibility profile and resistance mechanisms of these bacterial infections and their clinical associations on morbidity and mortality. We analyzed a retrospective cohort of burn patients. All patients included in this study had monobacterial blood stream infections during their hospital stay. We performed phenotypic and genotypic tests to determine the antimicrobial resistance mechanism and profile of each strain. Univariate and multivariate logistic regression analysis was performed between variables. We found 109 patients with monobacterial bacteremia. *Pseudomonas* spp. (50.7%), *A. baumannii* (46.4%), and *Klebsiella* spp. (13.8%) were the most common causative microorganisms. The *Pseudomonas* spp. isolates showed resistance to imipenem (81.5%), mainly by class A and class B carbapenemases. The *A. baumannii* isolates conferred resistance to imipenem (56.2%), mainly by class D carbapenemases. One quarter of *Klebsiella* spp. showed resistance to 3rd generation cephalosporins. We also observed that a total body surface area greater than 40% and three or more different types of invasive procedures might be related to increased mortality. Multidrug resistance is highly present. The extent of the burned area and a high number of different types of invasive procedures had an impact in decreasing survivorship in burn patients with bacteremia.

## 1. Introduction

Burn injuries are catastrophic illnesses, mostly associated with fire or scalding mechanisms and commonly linked to accidents, and they cause important social complications because more than half of the affected patients are economically active members of the population [1]. Given the complexity and extent of these lesions, the medical care of burn patients remains a challenging task. Clinical management requires a timely and multidisciplinary approach [2]. Previous studies have shown that the total body surface area (TBSA) affected by the burn injury is a determining factor in the host’s response and susceptibility to infection and thus overall survival [3,4]. Nearly 70% of cases had a TBSA < 10%, resulting in an overall mortality of 0.6% [1]; however, patients with a TBSA > 20% have greater intravascular volume depletion and depressed cardiac output, compromising perfusion to organs and tissues, in addition to the exacerbated inflammatory response originated by burn injury, factors which are associated with a higher rate of mortality [3,4].

Infections are the leading cause of death in burn patients [5]. This fatal complication has been explained because of the loss/alteration of skin layers and immunologic changes [6,7]. Infectious complications in patients with burn injuries are dictated by three factors: the source of infection, the mode of transmission (mainly associated with invasive procedures), and the host (e.g., skin barrier and cellular and humoral immunity) [8]. After the initial damage, burned surfaces are immediately more prone to infection. Colonization by different microorganisms occurs as early as 48 h, with a noticeable change in microbial composition and antimicrobial susceptibility [6]. A predominance of Gram-negative bacilli (GNB) infections has been associated with the second week of hospitalization [9,10]. *Acinetobacter baumannii*, *Klebsiella pneumoniae*, and *Pseudomonas aeruginosa* are the leading GNB causing healthcare associated infections with an important antimicrobial resistance association [11], they require a more complex and prolonged treatment, and are potentially transmitted to other patients [12]. Furthermore, an additional risk of infection has been observed in patients colonized by multidrug-resistant (MDR) microorganisms.

In this regard, bloodstream infection (BSI) is a common complication when considering burn wounds [3,4]. Tang et al. found that the isolates causing BSI in burn patients were *A. baumannii* (19.5%), *K. pneumoniae* (13.9%), and *P. aeruginosa* (9.3%) [11]. This last trend has been replicated in other studies [13,14], which has even shown an increase in BSI by GNB in this population [15]. As if this were not enough, Hu et al. reported multidrug-resistant (MDR) GNB strains and higher antimicrobial resistance rates in general for BSI in burn patients [14], which is extremely worrisome, considering that the treatment of MDR microorganisms is already challenging for clinicians, given the scarcity of therapeutic alternatives, the pharmacokinetic and pharmacodynamic challenges, and the delay/impossibility of starting appropriate antimicrobials [16].

Despite this evidence, little is known about the clinical associations and impact of GNB and MDR strains in burn patients. Therefore, we aimed to determine the antimicrobial susceptibility profile and resistance mechanisms of GNB, focusing on carbapenemases causing BSI in burn patients and evaluating the clinical associations and impact on mortality in this population.

## 2. Results

**Figure 1:**
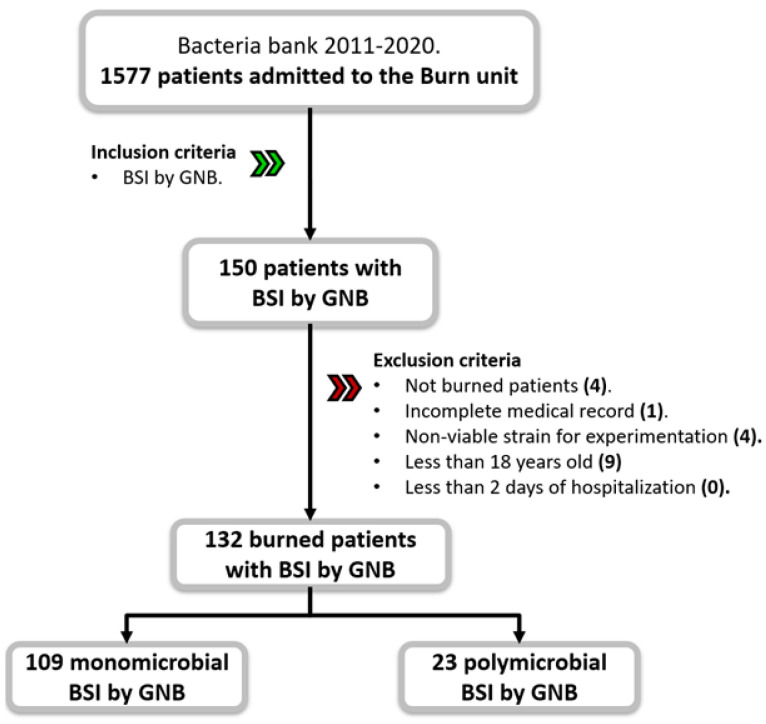
Flow diagram of study participants. The cohort was composed of 132 burn patients with BSI by GNB. Abbreviations: BSI—blood-stream infection; GNB—Gram-negative bacilli.

A total of 1577 patients were admitted to the burn unit during a 9-year study period (Figure 1). One hundred and fifty patients had BSI by GNB; consequently, 333 blood samples were processed by the Clinical Microbiology laboratory. A total of 18 patients were excluded; 9 patients were excluded because they were misclassified (4 were not burn patients; 1 had no record; in 4, the strains could not be recovered); and 9 were patients under 18 years of age, leaving a total of 132 patients and 109 monomicrobial isolates for analysis.

### 2.1. Demographics and Clinical Findings

Ninety-three patients (70%) were male with a median age of 35 (IQR, 25–49) years. Table 1 summarizes the main findings of this subsection. Nearly 95% of patients were referred from other health care facilities. These patients waited a median of 3 (IQR, 1–8) days after the burn event to be referred to our institution. The most common comorbidities identified at admission were overweight (41.7%) and obesity (34.1%). And their median LOS was 41 (IQR, 25–56) days. Third-degree burns (64.4%) caused by fire (75%) were common. Among the patients, 85.5% had a burned TBSA > 20%, and nearly one-third of patients had inhalation injury. The 97.7% required at least one invasive procedure (surgery or central venous catheter placement) before the onset of BSI. BSIs were confirmed at a median of 10.5 days after admission. Only 14 patients (10.6%) had a documented catheter-related bloodstream infection (CRBSI). Almost half (41.7%) had a soft tissue infection causing BSI; however, there was no other identified clinical infection that caused secondary bacteremia. We also observed other infectious complications in these patients, like fungemia by *Candida albicans* (9.1%) and *Clostridioides difficile* (3.8%) infections.

### 2.2. Microbial Findings and Antimicrobial Treatment

Eighty-two percent of BSI events were monomicrobial (Table 2). The 109 monomicrobial isolates were predominantly non-fermenting GNB (63.3%), with *Pseudomonas* spp. (50.7%) followed by *A. baumannii* (46.4%). In contrast, *Enterobacterales* represented 36.7% of the monomicrobial isolates, with a high predominance of *K. pneumoniae* (37.5%). MDR strains were observed in 66.1% of the monomicrobial BSIs, mostly consisting of non-fermenting GNB (55 strains; 79.7%, *p* < 0.001).

**Figure 2:**
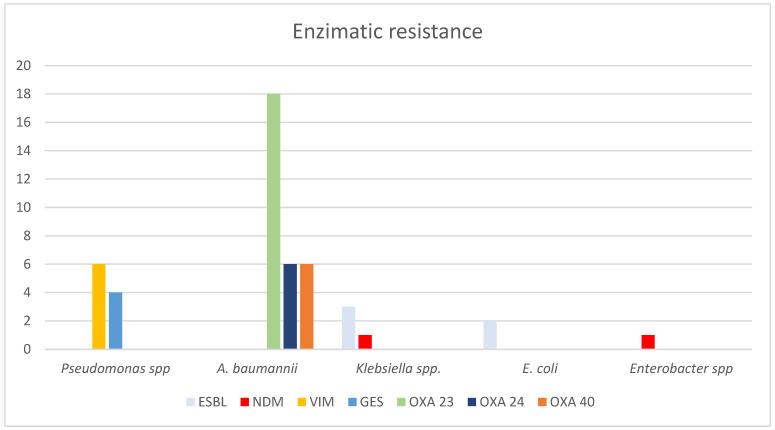
Mechanisms of resistance in the GNB causing BSI in burn patients. Abbreviations: ESBL—extended spectrum beta-lactamase; NDM—New Delhi metallo-β-lactamase; VIM—Verona integron-mediated metallo-β-lactamase; GES—Guiana extended-spectrum β-lactamase; OXA—carbapenem-hydrolyzing oxacillinase.

A high proportion of *Pseudomonas* spp. isolates were resistant to carbapenems (82.8% to IMP and 74.2% to MEM), quinolones (67.1%), and CAZ (62.8%). We detected *bla*_VIM_ and *bla*_GES_ in six and four strains, respectively (Figure 2). *A. baumannii* strains also showed high resistance rates to MEM (84.3%), with no resistance to aminoglycosides, COL, or TGC for this microorganism. However, *bla*_OXA-23-like_, *bla*_OXA-24-like_, and bla_OXA-40-like_ were identified.

On the other hand, *Klebsiella* spp. showed resistance to 3rd generation cephalosporins in more than 25% of isolates (33% to CAZ and 26% to FEP) and to quinolones (46%). Not all strains were intermediate to COL (13%). Extended spectrum β-lactamases (ESBL) were identified in three isolates. The antimicrobial susceptibility profiles of *E. coli* and *Enterobacter* spp. are shown in Table 2.

Empirical antimicrobial treatment was started within the first 24 h after a positive blood culture was obtained (Table 3). Based on the antibiogram, the prescription did not need to be changed in almost two-thirds of the monomicrobial BSIs (72 patients; 66%). Of concern was that non-fermenting GNB were more likely to require combined regimens (96.8%, *p* = 0.003), usually a carbapenem plus COL.

### 2.3. Clinical Associations for BSI by MDR Strains and Mortality

A subanalysis was carried out to identify factors associated with the development of bacteremia due to non-fermenting GNB, including 109 patients with monomicrobial infection, 40 patients (36.7%) with *Enterobacterales*, and 69 patients (63.3%) with non-fermenting bacteria. Patients with non-fermenting bacteremia presented a TBSA of >44.48% (*p* 0.029 (OR 2.44 95% CI 1.09–5.43)); their BSI origin was secondary (*p* 0.011 (OR 2.86 95% CI 1.26–6.48)); they required more use of vasopressors (*p* 0.005 (OR 3.20 95% CI 1.42–7.20)) and invasive mechanical ventilation (*p* 0.006 (OR 3.25 95% CI 1.4–7.57)) as well. In the multivariate analysis, only the secondary origin of BSI was identified as a variable of interest *p* 0.041 (adjusted OR 2.43 95% CI 1.03–5.69).

With all this information, a multivariate subanalysis was performed between patients affected by MDR and non-MDR bacteria (Table 4). This comparison showed that a delay of more than 3 days until admission to our institution (aOR 2.74 [1.13–6.63]; *p* = 0.025), a secondary BSI (aOR 2.80 [1.15–6.77]; *p* = 0.022), and having a non-fermenting GNB infection (aOR 4.13 [1.177–9.63]) were factors associated with the isolation of MDR strains in burn patients with BSI.

On the other hand, burn patients with BSI were more likely to die if the affected TBSA was >40% (OR 5.4 [2.04–12.93]; *p* = 0.001) and they required more than three different types of invasive procedures (OR 4.20 [1.59–11.12]; *p* = 0.004).

## 3. Discussion

Burn injuries are one of the most catastrophic forms of trauma. In patients with burns, it has been observed that sepsis secondary to bacteremia increases mortality [1]. Our study provided a comprehensive overview of BSI GNB in burn patients, which enabled us to make clinical inferences between clinical and microbiological data. Burn patients are usually men that belong to an economically active age group (median 35 years) [1]. Moreover, we found almost 87.12% of the population presented with a TBSA > 20%. This is worrisome, since the host’s response and risk for infection increases with more extensive TBSA [17,18,19]. Almost all patients had a central venous catheter placed; however, it was interesting to find a low CRBSI rate in our cohort. The incidence of CRBSI in burn patients varies widely worldwide. CRBSI incidence has been reported to be 7% in an 8-year study in China [13] and as low as 6.4% in an 11-year Turkish cohort [20]. In contrast, CRBSI incidence may be higher in Spain [10], Brazil [21], and Colombia [22], with 25.2%, 49%, and up to 72%, respectively. Certainly, CRBSI incidence is more likely to be high in short-term studies [22] and varies according to hospital level of care and laboratory techniques. On the other hand, secondary BSI was common in our cohort. This latter fact has been replicated by various research groups [10,23,24], but the most common infectious foci vary between surgical site infection in the burn area (37.7%) [23] and urinary tract infection (24.3%) [10]; in our study, soft tissue infection was the most common origin of bacteremia in 41.7%. It was not surprising to find soft tissues as the main foci in our cohort, considering the extensive TBSA reported; in fact, when it happened >40%, it was ultimately associated with secondary BSI by GNB in the bivariate analysis. Nevertheless, the foci may not be clearly identified in one third of the cases, as it has been noted by others [24]. Furthermore, the first positive blood culture was obtained in a 10-day median after admission to our institution. Previous authors have described an earlier onset of BSI by GNB in burn patients (first week) [6,7]; however, our findings are consistent with the time of wound colonization by GNB (second week), which has been extensively described in the literature [10,18,24,25,26]. The differences may be the result of external factors affecting the speed of colonization; some studies have highlighted the influence of the hospital environment and the frequent need for devices and/or surgical procedures for early colonization [6,7].

We found that the main infecting microorganisms were *Pseudomonas* spp., *A. baumannii*, and *Klebsiella* spp. Worldwide, *Pseudomonas* spp. has been reported as the most prevalent microorganism affecting burn patients with BSI [17,21,24,25]. In contrast, other cohorts have highlighted *A. baumannii* as the most important infecting microorganism [11]. It is important to note that most of these studies included both Gram-positive and Gram-negative microorganisms in their analysis, so the relative frequencies are likely to be higher [18,21,24,25]. It is of concern that our study identified more than half of the isolated strains as MDR, showing resistance to at least two or more groups of antibiotics, including cephalosporins, fluoroquinolones, aminoglycosides, carbapenems, and/or piperacillin/tazobactam [27]. Antimicrobial resistance is a global threat to healthcare systems. In fact, it is recognized that nearly five million deaths were associated with bacterial antimicrobial resistance in 2019 [15]. The growth in antimicrobial resistance rates in GNB bacteremia should be considered for adequate treatment as there has also been an increase in GNB resistance prevalence in the last decades in burn patients [15,17]. Among *Pseudomonas* spp. isolates causing BSI in burn patients, we found that nearly 85% of the strains were resistant to IMP. In contrast, resistance to carbapenems was detected in only 20% of strains in an Australian hospital in 2012 [24]. Like our findings, a study conducted in Spain from 2000 to 2014 found that 61% of isolates were resistant to IMP [10]. The same trend has been observed in China and Korea, with carbapenem resistance up to 75% and 95.9%, respectively [13,22]. Data from our country have confirmed a general resistance to MEM in 30% of the strains isolated from blood samples; however, not all these samples were obtained from burn patients [28]. Also, the INVIFAR network in our country analyzed blood cultures and found that 37.1% of *P. aeruginosa* isolates were resistant to carbapenems [29]. These latter studies pooled information from secondary and tertiary care units [29,30]. As a tertiary care unit admitting patients with previous exposure to a clinical setting, it is not surprising to find higher rates of antimicrobial resistance in our study. Antimicrobial resistance spreads easily; therefore, we were able to detect carbapenemases in *Pseudomonas* spp. isolates, mainly *bla*_VIM_ and *bla*_GES_ for *Pseudomonas* spp. strains. According to previous reports from the INVIFAR network, the most abundant gene in this microorganism was *bla*_IMP_ with 25.3%, followed by *bla*_VIM_ with 13% [29].

On the other hand, Patel et al. reported resistance to carbapenems in only 20% of *A. baumannii* strains [17]. This number may be as low as 6.8% according to Sousa et al. [10]. However, our study and similar research groups in Korea and China have found that resistance to carbapenems can be as high as 84.3%, 95.3%, and 95%, respectively [13,22]. Also, the most frequent resistance was mediated by the known OXA-like carbapenemases (*bla*_OXA-like 23_ and *bla*_OXA-like24_), like previous results in our country [28]. The story of *Enterobacterales* was similar to the GNB mentioned above. Spain reported that only 6.3% of *Klebsiella* spp. isolates were ESBL [10]; however, Korea and China identified nearly 80% of their strains as resistant to third-generation cephalosporins [13,22]. In Mexico, it is estimated that 80% of these bacterial groups have an ESBL phenotype [28,31]. Nevertheless, ESBL has been identified in a low proportion of *Enterobacterales*, and 30% of strains were resistant to third-generation cephalosporins in the USA according to the CDC [15].

Assessment of antimicrobial resistance was a key factor in our study and allowed us to determine whether the initial empirical antimicrobial regimen was appropriate to control the bacteria. Worldwide, approximately 30% of patients with BSI start an inappropriate empirical antibiotic regimen [32]; however, this number varies by nation and population type. In Israel, a higher proportion of patients (84%) started antimicrobials with an appropriate regimen [25], while a study in China found that this rate could be as low as 58.1% of patients receiving accurate empirical antimicrobial therapy [13]. We found that the initial empirical treatment was effective against the infecting GNB in 66.1%, which is lower than that reported in the general population. Even though inappropriate empirical therapy was not associated with an increase in mortality in this study, it is important to emphasize the relevance of providing adequate treatment.

We found that BSI by GNB were significantly associated with a TBSA > 44%, a condition likely related to the use of vasopressors and the need for mechanical ventilation, although they were not statistically associated with the development of BSI. An Australian study also found that MDR infection by GNB was associated with a TBSA > 20% and previous exposure to carbapenems [33]. Similarly, an increased number of different invasive procedures and a TBSA > 40% were related to higher mortality in these patients. In other studies, the presence of *Pseudomonas* spp. alone increased mortality by 30%; however, the presence of MDR isolates did not confer an additional risk of death [24,32,33,34]; we must bear in mind the severity of this pathology and that there are multiple variables that could impact the clinical outcome. Another important factor that should be mentioned that influences mortality is the delay that exists from the day of the burn until evaluation.

Our study has several limitations. First, the retrospective nature and single center of our study may be subject to differences in the collection, retrieval, recording, or handling of information. We may not have had specific important information, such as the exact time of the start of antibiotic treatment to evaluate mortality associated with delay. Second, our population is predominantly young male patients and therefore may not be generalizable to older patients; however, this is like other studies where burns tend to affect economically active men [1]. Third, additional mechanisms may mediate antimicrobial resistance in these strains (e.g., other carbapenems and efflux pumps). Despite this last fact, the mechanisms and genes targeted in our initial study design are the most common antimicrobial resistance mechanisms described in our country [28,31]. We did not perform pulse field electrophoresis studies to evaluate clonality and potential outbreaks that could be presented. Finally, our population was mainly composed of patients transferred from other institutions, where patients could have been colonized and later have developed the infectious disease at our hospital. It was worrisome but not surprising to find high rates of antimicrobial resistance, which differed from other studies performed in our country [28,31].

## 4. Materials and Methods

### 4.1. Study Design and Setting

We conducted a retrospective cohort study on burn patients treated between 2011 and 2020 at a tertiary care institution in Mexico City. The research was performed after approval by a local ethics committee with acceptance protocol number 88/19.

All burn patients included in this study were older than 18 years and experienced BSI with isolation of GNB, with signs and symptoms of infection classified as primary or secondary according to its origin [17]. Patients whose medical records were unavailable or incomplete (defined as lack of information, mainly without specification of antimicrobial treatment and/or when follow-up was stopped, without specifying the reason for discharge) were not included. In each case, the first bacterial isolate was selected for testing, and we also excluded those patients whose first clinical isolate from blood culture was not viable for antimicrobial testing. Our cohort was stratified according to the type of GNB (non-fermenting or enterobacterial), the detection of MDR strains, and death.

### 4.2. Data Collection

We retrospectively reviewed the electronic medical records for data collection. We retrieved demographic and clinical data (age and sex), comorbidities, length of stay (LOS), reason for discharge, burn type, TBSA, inhalation injury, invasive procedures, antimicrobial treatment, type of invasive procedures performed, BSI primary or secondary, and number of microorganisms recovered from blood culture. The LOS was recovered in days and was calculated based on the length of days between the BSI event and the subsequent hospitalization in our unit. On the other hand, the time to BSI presentation was expressed in days as the difference between arrival in our unit and the first positive blood culture. A central line-associated blood stream infection (CLABSI) was defined in a patient who had a central line within the 48 h period before the development of the BSI that was not bloodstream related to an infection at another site and a catheter-related blood stream infection (CRBSI) when, furthermore, the source of the BSI was associated to a positive catheter tip culture [17]. Relapse was defined when the same microorganism was identified in a subsequent episode, and reinfection was defined as the presence of different bacteria causing the same infection.

We also collected information of the invasive procedures performed. This variable was first recorded as dichotomous (presence/absence) for each type of invasive procedure (central venous catheter, orotracheal intubation, performance of at least one surgical procedure, etc.); it was then also recorded as a quantitative variable based on the sum of total of invasive procedures for each patient.

### 4.3. Strains Identification and Antimicrobial Susceptibility Profiles

Blood samples were inoculated into aerobic and anaerobic blood bottles (Becton Dickinson, Franklin Lakes, NJ, USA) and then incubated at 37 °C for 7 days in a semi-automated continuous monitoring system, Bactec (Becton Dickinson, USA). Positive samples were immediately processed for Gram staining and subcultured onto 5% sheep’s blood agar, MacConkey, chocolate, and phenylethyl alcohol supplemented with 5% sheep’s blood and dextrose Sabouraud agar; they were incubated at 37 °C aerobically (5% sheep’s blood and MacConkey) under 7.5% CO_2_ (chocolate) at 37 °C, anaerobically (phenylethyl alcohol agar with 5% sheep’s blood agar) at 37 °C, and dextrose Sabouraud agar at 30 °C, respectively. The initial identification and antimicrobial susceptibility profile were performed using the Vitek 2 Compact (BioMérieux, Craponne, France) according to the manufacturer’s instructions. The strains were then stored at −70 °C in a freezer (Revco Thermo Scientific, Waltham, MA, USA) until the start of carbapenemase evaluation. According to the susceptibility profile, MDR was defined if the strain showed resistance to at least two or more groups of antimicrobials evaluated in this study [27]. Once our eligible population was outlined, we thawed the GNB strains. To assess viability, we first inoculated these strains onto 5% sheep’s blood agar and incubated them at 37 °C for 48 h. Clinical strains were eliminated if no growth was observed after the second viability assessment. We then evaluated and confirmed the initial antimicrobial susceptibility profiles using the broth microdilution method according to Clinical & Laboratory Standards Institute (CLSI) recommendations, following M07-A10 and M100 guidelines [31,35]. The antimicrobials tested were amikacin (AK), ceftazidime (CAZ), cefepime (FEP), ciprofloxacin (CIP), colistin (CST), imipenem (IMP), levofloxacin (LVX), meropenem (MEM), piperacillin/tazobactam (TZP), and tigecycline (TGC) (all antibiotics were from Sigma Aldrich, USA). Experiments were performed in triplicate. *Acinetobacter baumannii* ATCC 19606, *Pseudomonas aeruginosa* ATCC 27853, and *Escherichia coli* ATCC 25922 were used as controls.

### 4.4. Carbapenemase Detection

All strains were tested for antimicrobial resistance mechanisms. To detect the presence of carbapenemases, the modified carbapenemase inactivation method (mCIM) and the carbapenemase inactivation method plus EDTA (eCIM) were performed as recommended [35]; briefly, the problematic bacterial strain was set into a tube with 2 mL of soy trypticase broth with a loop (1 μL loop for *Enterobacterales* and 10 μL loop for *P. aeruginosa*), and a disc of 10 μg meropenem was deposited into each tube, then the tubes were incubated at 37°/4 h.; Muller–Hinton plates were inoculated with 0.5 McFarland inoculum of *E. coli* ATCC 25922, and the meropenem discs were collocated onto these plates. We used *E. coli* ATCC 25922 as negative control; *K. pneumoniae* ATCC BAA-1705 was used as positive control. On the other hand, eCIM is a modification of the previous test that allows the identification of metallo-β-lactamases by the addition of EDTA at a final concentration of 5 mM; for this variant we used *Enterobacter cloacae* ATCC BAA-2468 as positive control.

We also assessed the presence of carbapenemase genes by endpoint polymerase chain reaction (PCR), where the obtained genetic material must subsequently be measured or examined by other types of physical analysis once the molecular reaction is finished. We used specific primers for the most common carbapenemase-encoding genes in our population [31]: Verona integron-mediated metallo-β-lactamase (VIM), imipenemase (IMP), New Delhi metallo-β-lactamase (NDM), *Klebsiella pneumoniae* carbapenemase (KPC), Guiana extended-spectrum β-lactamase (GES), and carbapenem-hydrolyzing oxacillinase (OXA): OXA-48-like, OXA-40-like, and OXA-2-like carbapenemase. The primer sequences used in this study are provided in the Supplementary Materials (Table S1). Briefly, one colony of each strain was used to extract chromosomal DNA using Chelex^®^ resin (Bio-Rad, Hercules, CA, USA), which as incubated at 96 °C for 20 min, then the tubes were centrifuged at 10,000 rpm, and the supernatant was separated into new tubes. The reaction mix for PCR was prepared with 10× buffer (Applied Biosystems, Foster City, CA, USA), with 1 mM MgCl_2_, 2 mM of each dNTP (Invitrogen, Carlsbad, CA, USA), 10 pmol of each targeting primer, 1.5 U Taq polymerase (Invitrogen, USA), and 5 μL of DNA. The final reaction volume was adjusted to 50 μL with free DNAse water. Amplification conditions were 1 cycle at 5 min/95 °C, 35 cycles (50 s/95 °C, 60 s/56 °C, and 50 s/72 °C), and 50 s followed by a final extension at 72 °C (Veriti Applied Biosystem, Waltham, MA, USA). Amplicons were visualized in a 1% agarose gel and run at 100 V (Gel Doc^TM^ XR+, Bio-Rad, USA) for 1 h. We used *A. baumannii* ATCC 19606, *E. cloacae* BAA-2468, and *K. pneumoniae* BAA 1705 as controls.

### 4.5. Statistical Analysis

Quantitative variables were expressed as mean and median with standard deviation (SD) and interquartile range (IQR), respectively. Normality was assessed by the Shapiro–Wilk test. Comparisons between independent variables were performed using the Mann–Whitney U or student t test, as appropriate, while categorical variables were expressed as absolute and relative frequencies. Fisher’s exact test or the chi-squared test were used to compare independent qualitative variables. The odds ratio (OR) was used to express the association of potential risk factors with MDR strains and mortality. When differences were found in those variables with a significance level at *p* < 0.05 in the bivariate analysis, the multivariate analysis was performed, and the Bonferroni correction was used for multiple comparisons. Two-sided 95% confidence intervals (CI) were reported. Statistical analysis was performed in STATA 14.0, and graphs were generated in GraphPad 7.0. All tests were two-tailed, and the significance level was set at *p* < 0.05.

## 5. Conclusions

BSI due to GNB is common in burn patients. BSI due to GNB occurs mainly after the first week of hospitalization and might be related to an increased risk of morbidity and mortality. We observed a high incidence of MDR microorganisms among our isolates; however, this condition did not increase mortality. Our study confirmed previous trends in which the extent of the TBSA and the number of different invasive procedures influenced mortality in this group of patients.

## Figures and Tables

**Table 1:** Demographic and clinical variables related to BSI by GNB in 132 burn patients.

Variable	Total
	*n* = 132 (%)
Sociodemographic data	
Sex, male	93 (70)
Age, median (IQR)	35.5 (25.5–49)
Referred patient	123 (93)
Comorbidities	
Glucose > 200 mg/dL (on admission)	11 (8.3)
Abnormal weight	102 (77.3)
Low weight	2 (1.5)
Overweight	55 (41.7)
Obese	45 (34.1)
Clinical presentation	
Type/mechanism of burn	
Fire	99 (75.0)
Electricity	25 (18.9)
Burned degree	
Second	47 (35.6)
Third	85 (64.4)
Inhalation injury	37 (28.0)
Days to hospital admission, median (IQR)	3 (1–8)
Days to isolation, median (IQR)	10.5 (5–20.5)
LOS, median (IQR)	41.5 (25.5–56.5)
TBSA, mean ± SD	44.9 ± 20.7
ABSI score < 8 points, ≥80% survival	61 (46.2)
SOFA, mean ± SD	5.5 ± 4.9
PITT, mean ± SD	4.4 ± 3.8
Infections and outcome	
Monomicrobial BSI	109 (82.6)
Primary BSI	70 (53.0)
CLABSI	56 (42.4)
CRBSI	14 (10.6)
Secondary BSI	62 (47.0)
Soft tissue	55 (41.7)
Reinfection	23 (17.4)
Relapse	19 (14.4)
Death	34 (25.8)
Candidemia	12 (9.1)
*C. difficile* infection	5 (3.8)
Invasive procedures	
Number of invasive procedures (types), median (IQR)	3 (2–3)
Surgery	129 (97.7)
Number of surgeries, median (IQR)	5 (3–8)
CVC	128 (97.0)
IMV	92 (69.7)
RRT	21 (15.9)

Abbreviations: BSI—blood-stream infection; GNB—Gram-negative bacilli; IQR—interquartile range; LOS—length of stay; TBSA—burned body surface area; ABSI—Abbreviated Burn Severity Index; SOFA—sequential organ failure assessment; PITT—PITT bacteremia score; CLABSI—central line-associated bloodstream infection; CRBSI—catheter-related bloodstream infection; CVC—central venous catheter; IMV—invasive mechanical ventilation; RRT—renal replacement therapy.

**Table 2:** Resistance profile of the most frequently isolated GNB.

Antimicrobial	*Pseudomonas* spp.		*A. baumannii*		*Klebsiella* spp.		*E. coli*		*Enterobacter* spp.	
	N	%	N	%	N	%	N	%	N	%
Amikacin	16	45.7	4	12	1	6.6	0	0	2	20
Ceftazidime	22	62.8	0	0	5	33.3	3	37.5	4	40
Cefepime	20	57.1	27	84.3	4	26.6	1	12.5	4	40
Ciprofloxacin	23	65.7	28	87.5	7	46.6	7	87.5	4	40
Levofloxacin	24	68.5	28	87.5	7	46.6	7	87.5	2	20
Imipenem	29	82.8	0	0	1	6.6	0	0	1	10
Meropenem	26	74.2	27	84.3	1	6.6	0	0	1	10
Piperacillin/Tazobactam	12	34.2	0	0	1	6.6	0	0	4	40
Colistin	1	2.8	0	0	2	13.3	0	0	0	0
Tigecycline	0	0	0	0	0	0	0	0	0	0

**Table 3:** Most frequently prescribed antimicrobial treatments.

	Total N (%)	Enterobacterales N (%)	Non-Fermenters N (%)	*p*
Type of Antimicrobial Treatment				
In-care antimicrobial therapy	102 (93.6)	39 (97.5)	63 (91.3)	>0.99
Accurate empirical treatment (first 24 h)	72 (66.1)	25 (62.5)	47/68.1)	0.551
Change/treatment upgrade	32 (29.4)	13 (32.5)	19 (27.5)	0.583
Monotherapy	20 (19.6)	13 (33.3)	7 (11.1)	0.004
Carbapenem	16 (80)	12 (92.3)	4 (57.1)	0.101
Cephalosporin	3 (15)	1 (77)	2 (28.6)	0.27
Quinolone	1 (50)	0	1 (14.3)	0.35
Combination	87 (85.3)	26 (66.7)	61 (96.8)	0.003
Two antimicrobials	61 (70.1)	18 (69.2)	43 (70.5)	0.906
Carbapenem and colistin	49 (80.3)	12 (66.7)	37 (86)	0.082
Three antimicrobials	15 (17.2)	6 (23.1)	9 (14.8)	0.347
Carbapenem, colistin, and rifampin	5 (5.7)	1 (3.8)	4 (6.6)	0.58
Four antimicrobials	9 (10.3)	2 (7.7)	7 (11.5)	0.719
Five antimicrobials	2 (2.3)	0	2 (3.3)	>0.99

**Table 4:** Univariate and multivariate analysis. Factors likely associated with the isolation of MDR GNB strains and mortality.

Variable	*p*	OR (95% CI)	*p*	aOR (95% CI)	*p*-Value
MDR isolation					
>3 days to burn unit admission	0.01	2.87 (1.28–6.43)	0.25	2.74 (1.13–6.63)	0.046
Secondary BSI	0.001	3.9 (1.70–8.90)	0.022	2.80 (1.15–6.77)	
CLABSI	0.001	0.26 (0.11–0.56)			
Soft tissue BSI	0.004	3.44 (1.47–8.03)			
Carbapenem and colistin	0.012	4.88 (1.42–16.82)			
Non-fermenter	<0.001	4.51 (2.03–9.98)	0.001	4.13 (1.77–9.63)	
Mortality					
Male	0.011	0.34 (0.15–0.78)			
Fire (mechanism of burn)	0.008	7.40 (1.66–32.86)			
Third degree burn	0.038	2.66 (1.05–6.70)			
>10 days to bacterial isolation	0.007	0.31 (0.13–0.72)			
>42 days of hospital stay	<0.001	0.18 (0.07–0.46)			
>1 BSI episode	0.017	2.72 (1.19–6.23)			
>3 invasive procedures (types)	0.004	4.20 (1.59–11.12)			
BSSA > 40%	0.001	5.4 (2.04–12.93)			

Abbreviation: OR—odds ratio; aOR—adjusted odds ratio; CI—confidence interval; MDR—multidrug resistant strain; BSI—blood-stream infection; CLABSI—central line-associated bloodstream infection; GNB—Gram-negative bacilli; BBSA—burned body surface area.

## Data Availability

Data supporting this study are included within the article and/or Supporting Materials.

## Supplementary Materials

The supporting information can be downloaded at: https://www.mdpi.com/article/10.3390/ijms251910458/s1.
