# Supplementary material for: Gram-Negative Bacilli Blood Stream Infection in Patients with Severe Burns: Microbiological and Clinical Evidence from a 9-Year Cohort
Source: Int J Mol Sci. 2024 Sep 28;25(19):10458. doi: 10.3390/ijms251910458 (PMC11476612; doi:10.3390/ijms251910458)
Supplement: Supplementary file 1 [file ijms-25-10458-s001.zip › ijms-3198192-supplementary.pdf]

Table S1. Primer sequences used to amplify carbapenemase encoding genes.

Gen	Sequence	Amplicon (bp)	Tm (°C)
bla$_{GES}$	5´-TCATTCACGCHCTATTVCTGGCA-3´<br>5´-CTATTTGTCCGTGCTCAGG-3´	857	56
bla$_{KPC}$	5´-ATGTCACTGTATCGCCGTCT-3´<br>5´-TTACTGCCCGTTGACGC-3´	798	56
bla$_{NDM}$	5´-ATGGAATTGCCCAATATT-3´<br>5’-TCAGYGCAGCTTGTCGGC-3´	650	56
bla$_{VIM}$	5´-AGATTGVCGATGGTGTTTGGT-3´<br>5’-GAGCAAGTCTAGACCGCCC-3´	430	56
bla$_{IMP}$	5´-GTTTATGTTCATACTTCGTTTG-3´<br>5´-CAACCAGTTTTGCHTTAC-3´	425	52
bla$_{OXA-48}$	5´-GAATGCCTGCGGTAGCAA-3´<br>5´-AAACCATCCGATGTGGGCAT-3´	438	56
bla$_{OXA-23}$	5´- GATCGGATTGGAGAACCAGA-3<br>5´- ATTTCTGACCGCATTTCCAT-3´	310	57
bla$_{OXA-24}$	5´- GGAATTCCATGAAAAAATTTATACTTCC-3´	400	57

	5´<br>CGGGATCCCGTTAAATGATTCCAAGATTTTCTA<br>GCG-3		
